# INPATIENT REHABILITATION AFTER ACUTE INJURY OR ILLNESS FOR PATIENTS WITH AND WITHOUT MENTAL HEALTH DISORDERS: SERVICE UTILIZATION, FUNCTIONAL IMPROVEMENT, AND PAIN

**DOI:** 10.2340/jrm.v58.45007

**Published:** 2026-09-07

**Authors:** Anja S. SUNDET, Solveig L. HAUGER, Ingrid A. HAVNES, Nada ANDELIC, Caroline USTVEDT, Grethe MÅNUM, Jeanette KLEVEN, Catrine BRUNBORG, Charles H. BOMBARDIER, Jennie PONSFORD, Marianne LØVSTAD

**Affiliations:** 1 Department of Research and Education, Sunnaas Rehabilitation Hospital, Bjørnemyr, Norway; 2 Department of Psychology, University of Oslo, Oslor, Norway; 3 Division of Mental Health and Addiction, Oslo University Hospital, Oslor, Norway; 4 Institute of Clinical Medicine, Faculty of Medicine, University of Oslo, Oslor, Norway; 5 Department of Physical Medicine and Rehabilitation, Oslo University Hospital, Oslor, Norway; 6 Center for Habilitation and Rehabilitation Models and Services (CHARM), Faculty of Medicine, Institute of Health and Society, University of Oslo, Oslor, Norway; 7 Department of Traumatic Brain Injury, Sunnaas Rehabilitation Hospital, Bjørnemyrr, Norway; 8 Beitostølen Healthsports Centre, Beitostølenr, Norway; 9 Department of Multitrauma, Neurology and Burns, Sunnaas Rehabilitation Hospital, Bjørnemyrr, Norway; 10 Oslo Center for Biostatistics and Epidemiology, Oslo University Hospital, Oslo, Norway; 11 Department of Rehabilitation Medicine, University of Washington, Seattle, Washington, USA; 12 Monash Epworth Rehabilitation Research Centre, School of Psychological Sciences, Monash University, Melbourne, Australia

**Keywords:** inpatient rehabilitation, mental health disorder, pain, opioid analgesics, post-acute, functional assessment

## Abstract

**Objective:**

Compare rehabilitation process and outcomes between patients with and without mental health disorders.

**Design:**

Observational cohort study.

**Subjects:**

130 patients receiving post-acute inpatient rehabilitation after acute injury or illness.

**Methods:**

Mental health disorders were assessed with diagnostic interviews. Adjusted regression analysis examined associations between mental health disorders and rehabilitation processes, functional improvement, pain, and patient satisfaction.

**Results:**

Patients with mental health disorders had more psychologist sessions (B = 5.87, 95% CI 3.87–7.87), more cancelled physiotherapy sessions (B = 1.15, 95% CI 0.38–1.93), multiprofessional team members collaborated more with external partners (B = 6.10, 95% CI 2.88–9.32), and nurses and team coordinators found their roles more demanding (OR = 5.47, 95% CI 1.38–21.76; OR = 7.43, 95% CI 2.21–24.94). Mental health disorders were associated with higher pain intensity and pain interference (B = 1.25, 95% CI 0.38–2.12; OR = 6.81, 95% CI 1.39–33.30). Opioid use was comparable on admission, but the mental health disorder group used more on discharge (OR = 4.5, 95% CI 1.75–11.55). Change in functional independence and patient satisfaction was comparable.

**Conclusion:**

Comorbid mental health disorders were associated with several aspects of the post-acute rehabilitation process, including resource use and pain management. These findings may inform the planning and organization of rehabilitation services for patients with mental health challenges.

## LAY ABSTRACT

This study examined whether and how having a comorbid mental health disorder is related to the inpatient rehabilitation process after an acute physical injury or illness. A total of 130 patients participated. Data were collected through medical charts, a trauma registry, diagnostic interviews with patients, and information from members of the multiprofessional teams. The results indicate that patients with mental health disorders report higher levels of pain, greater pain interference, and less tapering of opioids during inpatient rehabilitation compared with patients without comorbid mental health conditions. Having a mental health disorder was also associated with a need for closer follow-up from the rehabilitation team and more collaboration with external agencies. However, no difference between groups was found regarding functional outcomes or patient satisfaction, indicating equal profit from rehabilitation. Integrated mental health care and targeted interventions for pain management for patients with comorbid mental health disorders are warranted.

A large proportion of patients with physical injury and/or cognitive sequelae requiring specialized rehabilitation after physical injury or illness also present with comorbid mental health disorders (MHDs) and substance use disorders (SUDs) (1–3). These conditions often complicate the rehabilitation process. For example, substance use is a recognized risk factor for injury or illness (4–6), and psychological reactions may emerge during the recovery process following physical trauma. Mood disorders, anxiety disorders and SUDs are particularly common comorbid disorders across injury aetiologies (7, 8). This has also been confirmed in the same cohort as the current study, where a 38% mental health comorbidity rate was demonstrated. That study also showed that MHDs typically predate the physical injury or illness, as 72% of patients with an MHD during rehabilitation already had a pre-existing MHD at the time of injury or illness, whereas only 5% of those without a prior MHD developed one during rehabilitation (9). However, despite the well-documented high prevalence of comorbid MHDs in patients with injury and illness, the specific impact of mental health on the rehabilitation process, especially in the post-acute phase, remains poorly understood. One reason for this knowledge gap relates to the fact that patients with MHDs are often excluded from rehabilitation research (10), leaving medical practices and rehabilitation facilities unable to relate results from rehabilitation studies effectively to patients with these comorbidities. Of note, the National Institute for Health and Care Excellence (NICE) guidelines for rehabilitation after traumatic injury (11) recommend assessing psychological functioning in patients with complex rehabilitation needs to establish a baseline and to inform both initial interventions and future rehabilitation goals. However, due to the lack of evidence in this area, the guidelines emphasize the need for more research on the effects of mental health on early inpatient rehabilitation. Although few studies have examined the link between mental health and inpatient rehabilitation, functional performance has been shown to be negatively correlated with psychological variables (12). Furthermore, patients with spinal cord injuries and preinjury alcohol use problems have been shown to experience less functional improvement during inpatient rehabilitation (2). In addition, evidence from the long-term phase indicates that patients with MHDs and somatic injury represent a vulnerable patient group in need of special attention, as having an MHD is found to be associated with adverse long-term outcomes, including somatic complications and increased rehospitalization rates (13), lower return to work rates (14), nonadherence with medical treatment (15), greater use of pain medication (16), and a higher incidence of suicide (17). This complexity aligns with the biopsychosocial framework of the International Classification of Functioning, Disability, and Health (ICF) (18), which is a widely used conceptual framework in rehabilitation. Overall, there is a need for a better understanding of the influence of MHDs on inpatient rehabilitation processes and outcomes.

## Study aims

The main objective of this study was to examine whether, and in what ways, the presence of comorbid MHD is associated with the rehabilitation process and outcomes with regard to:

1. the rehabilitation process; number of treatment sessions, interdisciplinary collaboration, hospital service utilization, and participation in rehabilitation activities.
2. functional improvement; change in functional independence during the rehabilitation stay.
3. pain experience and management; experienced pain, pain interference with rehabilitation participation, and pain medication used on discharge.
4. patient satisfaction; self-reported satisfaction with their inpatient rehabilitation stays.

## METHODS AND MATERIALS

### Setting

The study was an observational prospective cohort study conducted at Sunnaas Rehabilitation Hospital (SRH), the largest specialized rehabilitation hospital in Norway, offering services to approximately half of the Norwegian population. SRH provides complex rehabilitation (Z50.80) in accordance with the International Classification of Diseases, 10^th^ Revision (ICD-10) (19) which requires the assistance of a multiprofessional team and at least 6 different health professions.

### Inclusion criteria and recruitment procedures

Participants were ≥ 18 years of age and admitted to inpatient rehabilitation at SRH due to injury or illness with acute onset. They were recruited consecutively from 3 clinical departments: (1) traumatic brain injury, (2) spinal cord injury, and (3) multi-trauma, neurology, and burns. All participants experienced complex rehabilitation needs. Eligible participants were approached by clinicians regarding participation as soon as it was considered ethically appropriate given their medical and emotional state, and written consent was secured by members of the research team. Patients with traumatic brain injury (TBI) were eligible for inclusion when able to provide informed consent, i.e., when they had regained orientation to personal circumstances, time and place as determined by the Galveston Orientation and Amnesia Test (20). The inclusion period spanned from January 2020 to February 2021, with a pause from March to the end of April 2020 due to the COVID-19 pandemic. Patients were recruited in close collaboration with treating physicians at SRH. Written informed consent was obtained from all participants. A flowchart of the inclusion process can be found in in Sundet et al. (9).

### Materials and measures

*Assessment of mental health and substance use disorders.* Mental health was assessed using the Norwegian version of the Mini International Neuropsychiatric Interview (M.I.N.I.), a brief semi-structured diagnostic interview based on ICD-10 criteria (21). The interviews were conducted by experienced clinical psychologists. For ethical reasons, the timing of the M.I.N.I. interview varied according to the patient’s psychological and somatic status during their stay. Information from medical charts concerning mental health was reviewed. Diagnostic decisions were made in consensus meetings within the research group after discharge, ensuring thorough differential diagnostic evaluation of each patient’s overall health status throughout their entire stay.

Patients who met the diagnostic criteria for at least 1 MHD during the inpatient rehabilitation stay at SRH were classified into the MHD group (*i*), and those without were classified into the non-MHD group (*ii*).

*Sociodemographic and medical data.* Patients were interviewed regarding demographic characteristics and asked to rate their financial worries during the past year on a numerical rating scale from 0 (no concern) to 10 (highest possible concern). A physician (author CU) extracted somatic ICD-10 codes from medical charts and classified aetiology of the illness/injury into 5 groups: spinal cord injury, acquired brain injury, multi-trauma (injuries to 2 or more body parts), polyneuropathy, and other diagnoses. For patients with traumatic injuries who received acute treatment at Oslo University Hospital Ullevaal, the Oslo University Hospital Trauma Registry provided information on injury severity scores. The Glasgow Coma Scale (GCS) (22) was used to assess level of consciousness on admission to the trauma centre. Scores range from 3 (unresponsive) to 15 (fully responsive). Overall injury severity was measured using the New Injury Severity Score (NISS) (23), derived from the Abbreviated Injury Scale (AIS-08) (24). NISS values range from 1 to 75, with scores greater than or equal to 9 indicating moderate-to-severe injury. Use of analgesics was recorded: (*i*) prior to the onset of injury or illness, as determined by the patient’s summary care record (a digital, national health system that disseminates health data across health services); (*ii*) on admission to SRH; and (*iii*) on discharge from SRH.

Additionally, analgesic use was categorized into: (*i*) mild opioids (codeine, tramadol), (*ii*) potent opioids (e.g., morphine, oxycodone), (*iii*) paracetamol and phenazone-caffeine, (*iv*) non-steroidal anti-inflammatory drugs (NSAIDs), and (*v*) other (gabapentin and pregabalin). Polypharmacy use was scored from 0 (no use) to 5 (use of all 5 analgesic groups).

*Functional independence.* The Functional Independence Measure (FIM) (25) was used to assess functional independence on admission and discharge from SRH. It evaluates 18 items (13 motor, 5 cognitive) on a 7-point scale, with higher scores indicating greater independence. The FIM has demonstrated excellent reliability and validity (26) across patient groups (27). Scores were extracted from medical charts and verified by at least 2 researchers. Total, motor, and cognitive scores are reported. A FIM-change score was used to measure change in functional status by subtracting FIM scores on admission from discharge scores. The average change in FIM scores per day, i.e., a FIM-efficiency score, was established by dividing the FIM change score by the number of days at SRH, in line with Bombardier and colleagues (2).

*The rehabilitation process.* Data on the rehabilitation process at SRH were extracted from medical records and included the number of attended and cancelled therapy sessions with each member of the multiprofessional team, the number of interdisciplinary team meetings without the patient, and the number of phone calls or meetings therapists had with external healthcare providers. Additional variables included participation in physical and occupational therapy group activities (yes/no), length of stay at SRH (in days), and whether the stay was extended (yes/no). On discharge, the team physiotherapist rated the patients’ adherence to their individual exercise programme as: (*I*) very good, (*II*) fairly good, or (*III*) low. These ratings were dichotomized into “high adherence” (very good or fairly good) and “low adherence”. The patients’ primary nurse and team coordinator assessed their roles in the rehabilitation process as: (*I*) less demanding, (*II*) average, or (*III*) more demanding than usual, which was dichotomized into “average” (less demanding or average) and “more demanding”.

Pain levels over the last week before discharge were rated from 0 (no pain) to 10 (worst pain imaginable), with scores above 3 being considered clinically relevant (28). Participants indicated whether pain affected their ability to follow the rehabilitation programme (yes/no). Satisfaction with the SRH stay was rated on a 5-point scale (1 = not at all, 5 = to a high degree) across 5 items: participation in rehabilitation decisions, tailored training, information for relatives, safety during the stay, and discharge process, which was summed into a total satisfaction score.

This study is a part of a larger project that includes a priori power estimates and is pre-registered on www.OSF.io, DOI number 10.17605/OSF.IO/MKWJD.

### Statistics

IBM SPSS, Version 30 (IBM Corp, Armonk, NY, USA), was used for statistical analyses. Sociodemographic and medical data were summarized using medians with interquartile ranges (IQR) (Q1–Q3) for continuous variables and proportions for categorical variables, unless stated otherwise. Patients were categorized into 2 groups: those with vs without a comorbid MHD. Group differences were assessed using the Mann–Whitney *U* test for continuous variables and χ^2^ or Fisher–Freeman–Halton exact tests for categorical variables. To examine the influence of MHD on rehabilitation outcomes, regression analyses were conducted. Six outcome variables were excluded due to insufficient variability (cell count < 5), leaving 23 variables to be analysed. For variables with cell count < 5, we report descriptive data only. Only complete case analyses were performed for multiple regression analyses, as only 3 of the outcome variables had missing values. Linear regression was used for continuous outcomes and logistic regression for categorical outcomes. Results are reported as unstandardized regression coefficients (B) or odds ratios (OR), along with 95% confidence intervals (CI), *p*-values, and explained variance (R² for linear models or Nagelkerke R² for logistic models). Multiple regression analyses were then conducted to determine whether MHD remained a significant predictor after adjusting for 6 potentially confounding factors: age, sex (dichotomizes as female (1) vs male (0), injury/illness mechanism (dichotomized as traumatic (1) vs non-traumatic (0), total Functional Independence Measure (FIM) score on admission, primary aetiology (dichotomized as acquired brain injury (1) vs other (0), and time from injury or illness to admission to specialized rehabilitation. These variables were selected based on observed between-group differences or their clinical and theoretical relevance to the outcomes. To account for multiple comparisons, the Benjamini–Hochberg procedure (29) was applied to control the false discovery rate (FDR), with a threshold of 0.10 (30). Adjusted *p*-values were ranked and compared with their corresponding Benjamini–Hochberg critical values. The largest *p*-value satisfying the condition *p* < (i/m) Q, along with all smaller *p*-values, was considered statistically significant. Adjusted *p*-values and their significance status (yes/no) are reported accordingly. Lastly, some post hoc analyses were not pre-specified and not adjusted for covariates.

## Results

### Sample characteristics

Of 216 patients considered for eligibility, 134 consented to participate. Four withdrew before assessment, resulting in a cohort of 130 participants, yielding a 77% inclusion rate. Most patients were male (69%), with a median age of 52 years (Table I). The majority (63%) had sustained a traumatic injury, all with NISS values 9 or higher, indicating moderate to severe injuries. Median length of stay at SRH was 66 days.

**Table I:** Sociodemographic and medical characteristics for the total sample and the 2 groups

Variables	All patients (*N* = 130)	MHD (*n* = 50)	Non-MHD (*n* = 80)	*p*-value
*Sociodemographic variables:*	Median [Q1–Q3], *n* (%)	Median [Q1–Q3], *n* (%)	Median [Q1–Q3], *n* (%)	
Age	52 [39-63]	49 [33–55]	58 [44–66]	0.001***
Sex: male/female	90 (69%) / 40 (31%)	28 (56%) / 22 (44%)	62 (78%)/18 (22%)	0.012**
*Relationship status at ToI:*				0.188
Married/cohabitating	76 (58%)	25 (50%)	51 (64%)	
Widowed/divorced/single	54 (42%)	25 (50%)	29 (36%)	
*Education level:*				0.369
≥ 13 years	71 (54%)	30 (60%)	41 (51%)	
< 13 years	59 (46%)	20 (40%)	39 (49%)	
*Source of income at time of injury/illness:*				<0.001***
Working or studying full-/part-time	64 (49%)	23 (46%)	41 (51%)	
Retirement pension	21 (16%)	2 (4%)	19 (38%)	
State-financed social benefits	42 (33%)	25 (50%)	17 (21%)	
Other sources of income	3 (2%)	0	3 (4%)	
Financial worries (NRS 0-10)	0 (0-4)	5 (0–7)	0 (0–2)	<0.001***
*Injury/illness related variables:*				
Time before admission to SRH (days)	29 [15-51]	41 [22–68]	21 [12–41]	<0.001***
Length of stay at SRH (days)	66 [45-93]	72 [49–94]	64 [41–92]	0.293
*Injury/illness mechanism:*				0.357
Non-traumatic	48 (37%)	21 (42%)	27 (34%)	
Traumatic	82 (63%)	29 (58%)	53 (66%)	
*Traumatic injury severity :*				
NISS^a^	26 [22-41]	28 [17–45]	25 [22–40]	0.480
GCS^b^	7 [4- 13]	14 [8–15]	14 [8–15]	0.449
*Main diagnosis:*				0.003**
Spinal cord injury	48 (36%)	10 (20%)	38 (48%)	
Acquired brain injury	33 (25%)	11 (22%)	22 (28%)	
Multitrauma	23 (18)	13 (26%)	10 (12%)	
Polyneuropathy	11 (9%)	7 (14%)	4 (5%)	
Other medical diagnoses^c^	15 (12%)	9 (18%)	6 (7%)	
*FIM scores on admission:*				
Physical	68 [41-81]	70 [44-80]	66 [38-81]	0.668
Cognitive	33 [30-35]	32 [29-34]	34 [30-35]	0.021**
Total	97 [72-111]	97 [73-111]	93 [70-111]	0.878

a New Injury Severity Score (NISS) scores were recorded for 64 of 82 trauma patients; 18 were missing owing to admissions to trauma centres other than Oslo University Hospital.

b Glasgow Coma Scale (GCS) scores were recorded for 25 of 28 patients with acquired brain injury; 3 scores are missing due to patients being admitted to other trauma centres.

c Other diagnoses include post-infectious disorders after COVID-19, amputations without multitrauma, radiculopathy, burn injuries, unexplained paralytic syndrome, muscular ischemia, and fractures without multitrauma.

MHD: mental health disorder; ToI: time of injury; SRH: Sunnaas Rehabilitation Hospital. Values in bold indicate statistically significant *p-*values*. p*≤0.05*, *p*≤0.025**, *p*<0.001***.

### Distribution of mental health disorders

Fifty of 130 patients (38%) met criteria for at least 1 MHD during inpatient rehabilitation. Of these, 52% (26/50) patients met the criteria for an anxiety disorder, with adjustment disorder being most common, followed by post-traumatic stress disorder and generalized anxiety disorder. In total, 44% (22/50) met criteria for a mood disorder, where recurrent depression was most common, followed by major depression and bipolar disorder. Furthermore, 32% (16/50) qualified for a SUD, where dependence or harmful use of alcohol were most common, followed by dependence on multiple psychoactive substances and opioid dependence. Overall, 44% (22/50) met the criteria for 2 or more MHDs. Further details regarding MHDs in this cohort have been reported in detail in our previous publication from the same cohort (9).

### Sociodemographic and medical differences between MHD and non-MHD groups

An overview of sociodemographic and injury-related variables for the total sample and each group is presented in Table I. Patients in the MHD group were significantly younger and had a higher proportion of women. A larger proportion of patients in the MHD group received state-financed social welfare benefits at the time of injury or illness and reported more financial worries. The interval from time of injury or illness to admission to SRH was longer for patients in the MHD group. On admission, cognitive FIM scores were lower in the MHD group, despite patients with MHDs not being overrepresented in the acquired brain injury group.

### Relationships between MHD and the rehabilitation process

When investigating the relationshiop between the presence of an MHD and the rehabilitation process, unadjusted analyses (Table II), identified 10 significant outcome variables. The following adjusted regression analyses confirmed that individuals with MHDs had more sessions with psychologists, more cancelled sessions with physiotherapists, and the multiprofessional teams had more phone calls/meetings with external healthcare agencies. Nurses were more than 5 times more likely to perceive their role as more demanding, and team coordinators were 7 times more likely to report increased role demands when caring for patients with MHD. All *p*-values were still significant when controlling for the 6 potential confounders (age, sex, injury aetiology, FIM-score, primary aetiology, and time from injury or illness) and remained significant after FDR correction (Table III).

**Table II:** Unadjusted regression analyses: association between mental health disorders and rehabilitation-related variables, functioning, pain experience and management, and patient satisfaction (*N* = 130)

Domains	Outcome variables	Beta coefficient (B)/odds ratio (OR)	95% CI	Nagelkerke R^2^	*p*-value
Rehabilitation process	*Number of attended sessions:*				
	Physician	0.55	(–1.12–2.20)	0	0.522
	On-call physician	1.15	(–0.41–2.71)	0.02	0.146
	Psychologist	1.26	(4.72–8.54)	0.27	<0.001***
	Physiotherapists	2.00	(–6.27–10.27)	0	0.633
	Occupational therapists	-2.51	(–9.24–4.21)	0	0.461
	Social worker	1.29	(0.24–2.33)	0.04	0.016**
	Speech therapist	-0.07	(–2.89–2.74)	0	0.959
	Clinical nutritionist	0.58	(–0.16–1.32)	0.02	0.122
	*Number of cancelled sessions:*				
	Physiotherapists	1.67	(0.90–2.44)	0.13	<0.001***
	Occupational therapists	0.64	(–0.15–1.43)	0.02	0.109
	*Interdisciplinary collaboration:*				
	Contact with external partners	7.46	(4.18–10.73)	0.14	<0.001***
	*Unplanned hospital treatment*				
	Extension of stay at SRH	0.50	(0.24–1.02)	0.04	0.057
	*Patient participation during rehabilitation:*				
	Participation in group activities with physiotherapist	1.38	(0.67–2.84)	0.01	0.379
	Participation in group activities with occupational therapist	1.46	(0.73–2.95)	0.01	0.287
	*Therapists’ evaluation:*				
	Adherence to individual exercise programme	3.26	(1.28–8.32)	0.68	0.014**
	Nurses’ evaluation of role	3.50	(1.35–9.11)	0.07	0.010**
	Team coordinator’s evaluation of role	3.73	(1.65–8.45)	0.1	0.002**
Functioning	*Change in functional independence:*				
	FIM total change score	-0.94	(–8.03–6.15)	0	0.794
	FIM total efficacy score	-0.06	(–0.15–0.04)	0.01	0.250
Pain experience & management	*Pain experience/ management:*				
	Pain levels (NRS 0–10)	1.60	(0.66–2.54)	0.09	0.001**
	Pain interference with rehabilitation	7.60	(2.07–27.90)	0.27	0.002**
	Opioid use on discharge	4.04	(1.88–8.69)	0.13	<0.001***
Patient satisfaction	*Patient satisfaction on discharge* ^a^	-0.56	(–1.55–0.44)	0.01	0.271

a Due to missing patient satisfaction scores for 15 patients, analysis was conducted on *n* = 115.

SRH: Sunnaas Rehabilitation Hospital; FIM: Functional Independent Measure; NRS: Numeric Rating Scale. Values in bold indicate statistically significant *p*-values. *p*≤0.05*, *p*≤0.025**, *p*<0.001***.

**Table III:** Adjusted regression analyses for the rehabilitation process domain with mental health disorders as independent variable while controlling for 6 potentially confounding variables

Outcome variables	Predictors	Beta coefficient (B) /odds ratio (OR)	95% CI	*p*-value	Significant after using an FDR of 0.1
*Number of attended sessions:*					
Psychologist	Mental health disorder	5.87	(3.87–7.87)	<0.001***	yes
	Age	–0.06	(–0.12––0.00)	0.042*	yes
	Sex	2.29	(0.34–4.24)	0.022**	yes
	FIM scores on admission	–0.07	(–0.11––0.03)	<0.001***	yes
	Injury/illness mechanism	–0.64	(–2.63–1.35)	0.526	
	Primary aetiology	–2.27	(–4.39––0.15)	0.036*	yes
	Time before admission to SRH (days)	0	(–0.04–0.03)	0.900	
	Adjusted R² = 0.36				
Social worker	Mental health disorder	1.02	(–0.07–2.11)	0.066	
	Age	–0.05	(–0.08––0.05)	0.004**	yes
	Sex	–0.26	(–1.32–0.81)	0.636	
	FIM scores on admission	–0.03	(–0.05––0.01)	0.005**	yes
	Injury/illness mechanism	0.30	(–0.78–1.39)	0.580	
	Primary aetiology	–1.69	(–2.85––0.53)	0.005**	yes
	Time before admission to SRH (days)	0	(–0.01–0.02)	0.632	
	Adjusted R² = 0.16				
*Number of cancelled sessions:*					
Physiotherapists	Mental health disorder	1.15	(0.38–1.93)	0.004*	yes
	Age	–0.01	(–0.03–0.02)	0.535	
	Sex	0.05	(–0.71–0.81)	0.901	
	FIM scores on admission	–0.03	(–0.04–0.01)	<0.001***	yes
	Injury/illness mechanism	0.52	(–0.26–1.29)	0.188	
	Primary aetiology	0.96	(0.13–1.79)	0.023**	yes
	Time before admission to SRH (days)	0.02	(0.01–0.04)	<0.001***	yes
	Adjusted R² = 0.28				
*Interdisciplinary collaboration:*					
Contact with external partners	Mental health disorder	6.10	(2.88–9.32)	<0.001***	yes
	Age	–0.11	(–0.21––0.02)	0.019**	yes
	Sex	–1.56	(–4.70–1.57)	0.326	
	FIM scores on admission	–0.19	(–0.25––0.13)	<0.001***	yes
	Injury/illness mechanism	0.75	(–2.45–3.95)	0.644	
	Primary aetiology	–1.54	(–4.95–1.88)	0.375	
	Time before admission to SRH (days)	0.06	(0.01–0.11)	0.029*	yes
	Adjusted R² = 0.33				
*Therapists’ evaluation:*					
Adherence to individual exercise programme	Mental health disorder	1.83	(0.60–5.59)	0.291	
	Age	0.99	(0.96–1.02)	0.470	
	Sex	0.81	(2.62–2.50)	0.714	
	FIM scores on admission	0.97	(0.95–1.00)	0.017**	
	Injury/illness mechanism	1.61	(0.47–5.53)	0.448	
	Primary aetiology	1.34	(0.36–5.27)	0.645	
	Time before admission to SRH (days)	1.03	(1.01–1.05)	<0.001***	yes
	Nagelknerke R² = 0.29				
Nurses’ evaluation of role	Mental health disorder	5.47	(1.38–21.76)	0.016**	yes
	Age	0.99	(0.96–1.03)	0.603	
	Sex	1.21	(0.39–3.73)	0.742	
	FIM scores on admission	0.95	(0.93–0.98)	<0.001***	yes
	Injury/illness mechanism	2.16	(0.61–7.62)	0.230	
	Primary aetiology	5.36	(0.90–31.81)	0.640	
	Time before admission to SRH (days)	1.01	(0.99–1.02)	0.600	
	Nagelknerke R² = 0.37				
Team coordinator’s evaluation of role	Mental health disorder	7.43	(2.21–24.94)	0.001**	yes
	Age	1.01	(0.98–1.04)	0.700	
	Sex	0.32	(0.11–0.96)	0.041*	yes
	FIM scores on admission	0.96	(0.93–0.97)	<0.001***	yes
	Injury/illness mechanism	1.31	(0.44–3.90)	0.623	
	Primary aetiology	2.20	(0.59–8.20)	0.240	
	Time before admission to SRH (days)	1.01	(1.00–1.03)	0.120	
	Nagelknerke R² = 0.40				

FIM: Functional Independent Measure; SRH: Sunnaas Rehabilitation Hospital. Values in bold indicate statistically significant *p*-values. *p*≤0.05*, p≤0.025**, p<0.001***.

Patientes with MHDs had more sessions with the social workers and lower adherence to their individual exercise programme in the unadjusted analysis, but these findings were no longer significant in the adjusted analysis. Due to low variability, analyses were not possible for all selected outcomes, but descriptive analyses revealed interesting trends: Patients with MHDs cancelled twice as many appointments with psychologists and speech therapists, compared with the non-MHD group Patients with MHDs also cancelled appointments with the physician and had higher cancellation rates for sessions with social workers and clinical nutritionist, compared to the non-MHD group (Appendix S1, Table I). These results are descriptive only and should be interpreted with caution.

### Association of mental health disorders with functional improvement

The presence of an MHD was not associated with FIM change scores or FIM efficacy scores in the unadjusted models, neither for total scores nor subscales (Table II). However, descriptive analysis showed a difference between the MHD and non-MHD groups regarding FIM cognitive scores on admission (Table I). To further explore this, a post hoc analysis of the 5 items that comprise the cognitive subscale was conducted. This showed that participants with MHDs had lower scores related to social interaction both on admission (5 vs 7, B = –0.940, 95% CI –1.456 to –0.424, R^2^ = 0.09, *p* **<** 0.001), and on discharge (6 vs 7, B = –0.965, 95% CI –1.270 to –0.660, R^2^ = 0.23, *p* **<** 0.001), and lower scores on problem-solving capacity on discharge (6 vs 7, B = –0.598, 95% CI –0.971 to –0.224, R^2^ = 0.07, *p* = 0.002), (see Table SII for details). These *post hoc* analyses are unadjusted and should be interpreted with caution.

### Association of mental health disorders with pain and pain medication use

The association of MHDs with pain was clear, as patients with MHDs reported significantly more pain, were 6 times more likely to report that pain interfered with their rehabilitation during the last week before discharge and had a 4.5-fold increased likelihood of using opioids on discharge compared with the non-MHD group. These associations remained significant after adjusting for the 6 potentially confounding factors, and also after FDR corrections (Table IV).

**Table IV:** Adjusted regression analyses for pain experience and pain management with mental health disorders as independent variable while controlling for 6 potentially confounding variables (*N* = 130)

Outcome variables	Predictors	Beta coefficient (B)/odds ratio	95% CI	*p*-value	Significant after using an FDR of 0.1
*Pain experience:*					
Pain levels on discharge (NRS 0-10)^a^	Mental health disorder	1.25	(0.38-2.12)	0.005*	yes
	Age	0.03	(0.01-0.06)	0.019**	yes
	Sex	1.44	(0.57-2.31)	0.001**	yes
	FIM scores on admission	-0.002	(-0.02-0.01)	0.843	
	Injury/illness mechanism	-0.66	(-1.54-0.23)	0.137	
	Primary aetiology	2.13	(1.22-3.05)	<0.001***	yes
	Time before admission to SRH (days)	0.01	(-0.01-0.02)	0.445	
	Adjusted R² = 0.36				
*Pain management:*					
Pain interference with rehabilitation^b^	Mental health disorder	6.81	(1.39-33.30)	0.018**	yes
	Age	0.97	(0.92-1.03)	0.376	
	Sex	0.60	(0.14-2.58)	0.489	
	FIM scores on admission	0.99	(0.96-1.03)	0.721	
	Injury/illness mechanism	2.05	(0.41-10.11)	0.380	
	Primary aetiology	4.86	(0.22-106.10)	0.315	
	Time before admission to SRH (days)	1.01	(0.99-1.04)	0.397	
	NagelkerkeR² = 0.33				
Opioid use at discharge	Mental health disorder	4.50	(1.75-11.55)	0.002**	yes
	Age	1.01	(0.98-1.04)	0.539	
	Sex	1.60	(0.69-3.74)	0.273	
	FIM scores on admission	0.99	(0.98-1.01)	0.457	
	Injury/illness mechanism	0.99	(0.41-2.44)	0.999	
	Primary aetiology	3.99	(1.25-12.72)	0.019**	yes
	Time before admission to SRH (days)	1.00	(0.98-1.01)	0.820	
	Nagelkerke R² = 0.23				

a Due to missing pain level scores on discharge for 13 patients, analysis was conducted with *n*: 117.

b Analysis were conducted with *n*: 48 as 13 patients had missing pain level scores and only 48 had NRS pain scores above 3 (defined as clinically relevant pain).

NRS: Numeric Rating Scale; SRH: Sunnaas Rehabilitation Hospital; FIM: Functional Independent Measure. Values in bold indicate statistically significant *p*-values.

*p*≤0.05*, *p*≤0.025**, *p*<0.001***.

**Fig. 1:**
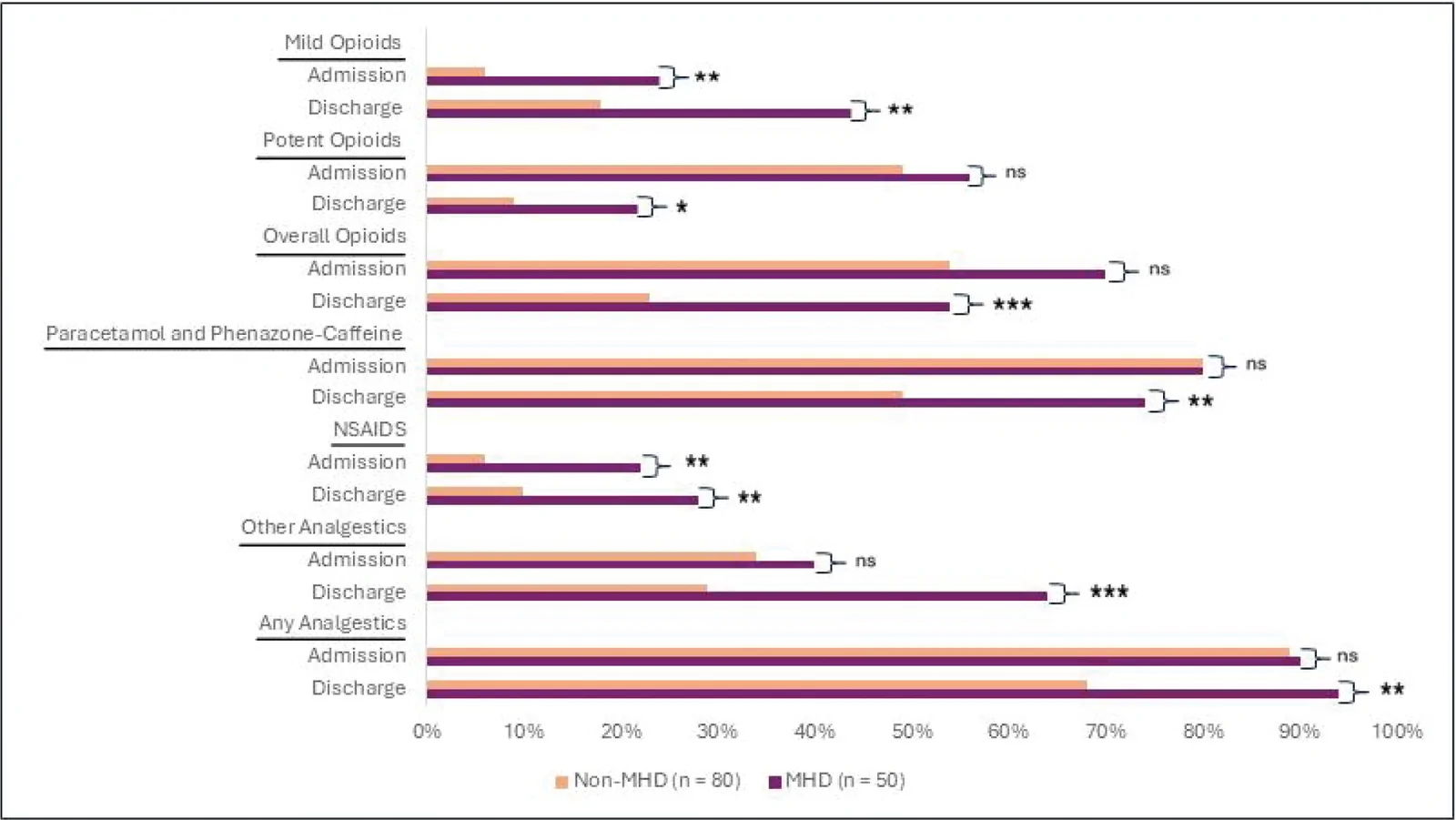
Between-group differences in analgesic use on admission and discharge from the rehabilitation facility in patients with mental health disorders (*n* = 50) vs those without (*n* = 80).

Post hoc analyses were performed to determine whether the difference in opioid use on discharge was also present on admission to SRH and are shown in Fig. 1 (see Table SIII and SIV for details). These *post hoc* analysis showed no significant between-group differences in terms of overall opioid use or potent opioid use at the time of injury or illness, nor upon admission to rehabilitation. However, use of mild opioids was significantly higher in the MHD group on admission (24% vs 6%, OR = 4.74, 95% CI 1.56–14.43, *p* = 0.006) and this difference remained present on discharge (44% vs 18%, OR = 3.70, 95% CI 1.66–8.27, *p* = 0.001). We also explored the use of other analgesics and found that the MHD group had higher overall analgesic consumption on discharge compared with those without MHDs (94% vs 68%, OR = 7.54, 95% CI 2.15–26.52, *p* = 0.002). Furthermore, the MHD group exhibited twice the use of polypharmacy on discharge (2.32 vs 1.14, B = 1.18, 95% CI 0.781–1.584, R^2^ = 0.21, *p*** <** 0.001) (Table SIV). These post hoc analyses are unadjusted and must be interpreted with caution. In 2 cases, opioid use was related to premorbid prescription of opioid agonist treatment.

Additionally, differences in opioid usage patterns were evident, with 30% (15/50) of individuals in the MHD group using opioids daily on discharge, compared with 6% (5/80) in the non-MHD group (OR = 6.43, 95% CI 2.16-19.10, *p* < 0.001).

### Patient satisfaction with their rehabilitation stay

On discharge, patients with MHDs reported comparable satisfaction with their rehabilitation stay to those without MHDs (Table II).

## DISCUSSION

This study found that patients with MHDs during inpatient rehabilitation had more sessions with psychologists, cancelled more sessions with physiotherapists, reported greater pain and pain interference, and discontinued opioid use to a lesser degree. The care of patients with MHDs also incolved more interdisiplinary collaboration, and was experienced as more demanding for nurses and team coordinators, but func-tional independence gains and treatment satisfaction were comparable to those without MHDs

### Association between mental health disorders and rehabilitation process

This study is among the first to indicate that comorbid MHDs are associated with the rehabilitation processes and resource requirements, including an increased need for collaboration and coordination within multiprofessional teams and with external agencies. Patients with MHDs appeared to require greater flexibility from the team, reflected in psychological challenges, reduced problem-solving capacity, and higher cancellation rates for therapy sessions. Rehabilitation of patients with MHDs thus may require additional effort from the team. Nurses play a crucial role in creating a supportive environment for patients, but may perceive a lack of sufficient value for their team contributions (31). This study indicates that a patient’s mental health status is strongly associated with nurses’ perceptions of work demands, and that coordinating care for patients with MHDs also appears challenging, given the high demands perceived by team coordinators. However, their satisfaction with their rehabilitation stay suggests that the rehabilitation teams were able to accommodate their needs.

On admission to rehabilitation, patients with MHDs had longer time since injury or illness. Whether this was related to a selection bias with regard to early access to rehabilitation services or to medical issues remains unknown. In contrast to 1 prior study (2) where patients with spinal cord injuries and a history of alcohol use problems showed weaker functional improvement during inpatient rehabilitation compared with those without, the current study showed no differences in functional improvement between the MHD and non-MHD groups, indicating that they profited as much as their non-MHD peers from rehabilitation. However, patients with MHDs elicited more follow-up support and more interdisciplinary collaboration, which may have contributed to greater functional improvement. According to the ICF, function is the result of positive interactions between an individual with a health condition and contextual factors, while disability denotes the negative aspects of this interaction. Hence, environmental factors can act as both barriers and facilitators of functioning. The comparable FIM efficacy scores between patients with and without MHDs might have been influenced by the high level of psychosocial support provided to patients with MHDs. Additionally, the current study included a heterogeneous patient group in respect of both injury aetiologies and MHDs, compared with prior research (2) that focused exclusively on spinal cord injury and alcohol use, in the United States vs Norway, factors that may all have contributed to diverging results.

The bidirectional relationship between emotional distress and pain is well established (33, 34), yet not fully understood (35). Interestingly, although several factors such as sex, age, and primary aetiology were associated with pain experience in the current study, having an MHD was the only variable that showed a statistically significant association with whether patients reported that pain interfered with rehabilitation. In future studies, factors such as pain catastrophizing (36) and fear avoidance (37) should be investigated.

For patients with concurrent pain and MHDs during post-acute rehabilitation, guidelines (11) and clinical studies (38) emphasize that interventions should target emotional functioning and pain concurrently.

An important finding was that patients with MHDs had more than 4 times higher risk of using opioids on discharge compared with the non-MHD group, despite no difference in overall opioid use on admission or at the time of injury or illness. Although potent opioid use was reduced, mild opioid use had increased in the MHD group on discharge. Slower tapering of opioids during rehabilitation is a strong candidate in explaining the group difference. Some individuals with MHD may use opioids to cope with emotional distress (39). Thus, an elevated risk of opioid dependence and overdose is concerning, as physical injuries, poorly managed pain, and the presence of MHDs are common pathways to long-term use of opioids (40). Daily use of prescribed opioids is also associated with a twofold higher risk of long-term opioid use compared with occasional use (16), and mild opiates contribute as much as potent opioids to long-term use (41). However, slow tapering of opioids may be appropriate for some patients, particularly those with long-term use or high pain levels, as gradual dose reduction can help minimize withdrawal symptoms.

### Strengths and limitations

Several limitations should be mentioned. Although we have demonstrated associations between MHD and several outcomes, we cannot draw causal conclusions or rule out that factors such as personality traits, motivation, or medical complications might have contributed to the associations. Also, subacute rehabilitation is a highly individualized process making the use of non-standardized terms such as “more” and “less than usual” challenging. Additionally, the questions used to assess role demands and adherence to individual exercise programmes are subjective and not validated therapist reports, with unknown inter-rater reliability, where individual differences in response style cannot be ruled out. Also, we included a heterogeneous MHD group, and the limited sample size precluded further subcategorization by type of MHDs.

This study is primarily descriptive in nature and does not contain outcome measures where minimal clinically importance difference has been established. However, we do find that significant associations described in this study between MHD and various rehabilitation outcomes is of potential clinical interest, but in need of replication. Although FIM is a validated instrument, it may still not be sufficiently sensitive to capture nuanced change, particularly in the cognitive and psychosocial domains, which may have influenced the lack of group differences.

Finally, specialized rehabilitation places high demands on patients’ active participation. We cannot exclude the possibility of referral bias, where some patients with severe mental illnesses were not referred to SRH. However, no referred patients were excluded due to psychosis. In addition, patients with cognitive or developmental impairments affecting their ability to provide informed consent, or those requiring transfer to other facilities before inclusion was considered ethically appropriate, may not have been enrolled, potentially introducing recruitment bias towards less severely affected patients. At SRH, psychology services are embedded in all service provision. The findings therefore do not necessarily generalize to other countries and different healthcare systems.

Lastly, the study was conducted during the COVID-19 pandemic, which may have had an impact on mental stress, prioritization, and resource utilization. However, meta-analyses have found overall low change estimates when comparing mental health symptoms before and during the COVID-19 pandemic, especially beyond the first months (42), and our data collection was paused in the early months of the pandemic. Also, although SRH did not reduce post-acute rehabilitation bed capacity during the pandemic, admissions may nevertheless have been affected by the prioritization of patients with moderate to severe COVID-19 (32), some of whom were included in this study. Although SRH closed its outpatient services during the pandemic in line with most European cities (32), the post-acute wards remained open with normal staffing and between-group comparisons were made on equal terms regarding COVID-19 effects.

Despite these limitations, however, this study is to our knowledge one of few to explore associations between comorbid MHDs and the rehabilitation process and outcomes. Broad access to data, use of diagnostic interviews, and interdisciplinary diagnostic consensus are strengths of the study.

### Clinical implications and future directions

Comorbid MHDs were associated with several aspects of the post-acute rehabilitation process, particularly related to resource use and pain management. Treatment strategies to jointly address pain and mental health challenges during post-acute rehabilitation are warranted to prevent chronic pain and long-term opioid use in this group. Future studies should include larger samples to explore differences between specific diagnoses or diagnostic categories, as well as between patients with pre-existing and newly developed disorders.

## References

[1] Meroni R, Beghi E, Beghi M, Brambilla G, Cerri C, Perin C, et al. Psychiatric disorders in patients suffering from an acute cerebrovascular accident or traumatic injury, and their effects on rehabilitation: an observational study. Eur J Phys Rehabil Med 2013; 49: 31–39. 23138676

[2] Bombardier CH, Stroud MW, Esselman PC, Rimmele CT. Do preinjury alcohol problems predict poorer rehabilitation progress in persons with spinal cord injury? Arch Phys Med Rehabil 2004; 85: 1488–1492. 10.1016/j.apmr.2003.10.010 15375822

[3] Gould KR, Ponsford JL, Johnston L, Schonberger M. The nature, frequency and course of psychiatric disorders in the first year after traumatic brain injury: a prospective study. Psychol Med 2011; 41: 2099–2109. 10.1017/S003329171100033X 21477420

[4] Brathen CC, Jorgenrud BM, Bogstrand ST, Gjerde H, Rosseland LA, Kristiansen T. Prevalence of use and impairment from drugs and alcohol among trauma patients: a national prospective observational study. Injury 2023; 54: 111160. 10.1016/j.injury.2023.111160 37944451

[5] Tetrault M, Courtois F. Use of psychoactive substances in persons with spinal cord injury: a literature review. Ann Phys Rehabil Med 2014; 57: 684–695. 10.1016/j.rehab.2014.10.002 25455026

[6] Tollisen KH, Bjerva M, Hadley CL, Dahl GT, Hogvall LM, Sandvik L, et al. Substance abuse-related admissions in a mixed Norwegian intensive care population. Acta Anaesthesiol Scand 2020; 64: 329–337. 10.1111/aas.13506 31721148

[7] Herrera-Escobar JP, Seshadri AJ, Stanek E, Lu K, Han K, Sanchez S, et al. Mental health burden after injury: it’s about more than just posttraumatic stress disorder. Ann Surg 2021; 274 :e1162–e1169. 10.1097/SLA.0000000000003780 32511129

[8] Thom R, Silbersweig DA, Boland RJ. Major depressive disorder in medical illness: a review of assessment, prevalence, and treatment options. Psychosom Med 2019; 81: 246–255. 10.1097/PSY.0000000000000678 30720699

[9] Sundet AS, Lovstad M, Havnes IA, Andelic N, Ustvedt C, Manum G, et al. Rates and temporal onset of mental health disorders during inpatient rehabilitation after acute physical injury or illness: an observational cohort study. PLoS One 2025; 20: e0338207. 10.1371/journal.pone.0338207 41474681 PMC12755833

[10] Van Spall HG, Toren A, Kiss A, Fowler RA. Eligibility criteria of randomized controlled trials published in high-impact general medical journals: a systematic sampling review. JAMA 2007; 297: 1233–1240. 10.1001/jama.297.11.1233 17374817

[11] National Institute for Health and Care Excellence NICE. Rehabilitation After Traumatic Injury 2020 [cited 2025 Apr 15]. Available from: www.nice.org.uk/guidance/ng211 35471781

[12] Lee W, Jeong S, Lee B-S, Lim J-C, Kim O. Association between functional outcomes and psychological variables in persons with spinal cord injury. Sci Rep 2023; 13: 23092. 10.1038/s41598-023-50252-8 38155215 PMC10754915

[13] Zatzick DF, Rowhani-Rahbar A, Wang J, Russo J, Darnell D, Ingraham L, et al. The cumulative burden of mental, substance SSE, and general medical disorders and rehospitalization and mortality after an injury. Psychiatr Serv 2017; 68: 596–602. 10.1176/appi.ps.201600311 28142384 PMC5550030

[14] Gabbe BJ, Simpson PM, Harrison JE, Lyons RA, Ameratunga S, Ponsford J, et al. Return to work and functional outcomes after major trauma: who recovers, when, and how well? Ann Surg 2016; 263: 623–632. 10.1097/SLA.0000000000001564 26779977

[15] DiMatteo MR, Lepper HS, Croghan TW. Depression is a risk factor for noncompliance with medical treatment: meta-analysis of the effects of anxiety and depression on patient adherence. Arch Intern Med 2000; 160: 2101–2107. 10.1001/archinte.160.14.2101 10904452

[16] Clark JM, Cao Y, Krause JS. Risk of pain medication misuse after spinal cord injury: the role of substance use, personality, and depression. J Pain 2017; 18: 166–177. 10.1016/j.jpain.2016.10.011 27836813

[17] Asheim A, Nilsen SM, Svedahl ER, Kaspersen SL, Bjerkeset O, Janszky I, et al. Risk of suicide after hospitalizations due to acute physical health conditions: a cohort study of the Norwegian population. BMC Med 2024; 22: 396. 10.1186/s12916-024-03623-5 39285471 PMC11406799

[18] World Health Organization (WHO). International Classification of Functioning, Disability and Health (ICF) 2001 [cited 2026 Apr 10]. Available from: www.who.int/standards/classifications/international-classification-of-functioning-disability-and-health

[19] World Health Organization (WHO). International Statistical Classification of Diseases and Related Health Problems (ICD). 12th ed. Geneva: World Health Organization; 1992.

[20] Levin HS, O’Donnell VM, Grossman RG. The Galveston Orientation and Amnesia Test: a practical scale to assess cognition after head injury. J Nerv Ment Dis 1979; 167: 675–684. 10.1097/00005053-197911000-00004 501342

[21] Sheehan DV, Lecrubier Y, Sheehan KH, Amorim P, Janavs J, Weiller E, et al. The Mini-International Neuropsychiatric Interview (M.I.N.I.): the development and validation of a structured diagnostic psychiatric interview for DSM-IV and ICD-10. J Clin Psychiatry 1998; 59: 22–33. 9881538

[22] Teasdale G, Jennett B. Assessment of coma and impaired consciousness: a practical scale. Lancet 1974; 2: 81–84. 10.1016/s0140-6736(74)91639-0 4136544

[23] Osler T, Baker SP, Long W. A modification of the injury severity score that both improves accuracy and simplifies scoring. J Trauma 1997; 43: 922–925. 10.1097/00005373-199712000-00009 9420106

[24] Association for the Advancement of Automotive Medicine. The abbreviated injury severity scale (AIS) 2005 [cited 2025 May 18]. Available from: http://www.aaam.org/abbreviated-injury-scale-ais/

[25] Linacre JM, Heinemann AW, Wright BD, Granger CV, Hamilton BB. The structure and stability of the functional independence measure. Arch Phys Med Rehabil 1994; 75: 127–132. 8311667

[26] Hamilton BB, Laughlin JA, Fiedler RC, Granger CV. Interrater reliability of the 7-level functional independence measure (FIM). Scand J Rehabil Med 1994; 26: 115–119. 7801060

[27] Heinemann AW, Kirk P, Hastie BA, Semik P, Hamilton BB, Linacre JM, et al. Relationships between disability measures and nursing effort during medical rehabilitation for patients with traumatic brain and spinal cord injury. Arch Phys Med Rehabil 1997; 78: 143–149. 10.1016/s0003-9993(97)90255-0 9041894

[28] Wisloff-Aase K, Raeder J, Manum G, Lovstad M, Schanke AK, Dyb G, et al. Chronic pain among the hospitalized patients after the 22 July 2011 terror attacks in Oslo and at Utoya Island. Acta Anaesthesiol Scand 2019; 63: 913–922. 10.1111/aas.13373 30968401

[29] Benjamini Y, Hochberg Y. Controlling the false discovery rate: a practical and powerful approach to multiple testing. J Roy Stat Soc 1995; 57: 289–300. 10.1111/j.2517-6161.1995.tb02031.x

[30] Hellstrom T, Andelic N, de Lange AG, Helseth E, Eiklid K, Westlye LT. Apolipoprotein varepsilon4 status and brain structure 12 months after mild traumatic injury: brain age prediction using brain morphometry and diffusion tensor imaging. J Clin Med 2021; 10: 418. 10.3390/jcm10030418 33499167 PMC7865561

[31] Long AF, Kneafsey R, Ryan J. Rehabilitation practice: challenges to effective team working. Int J Nurs Stud 2003; 40: 663–673. 10.1016/s0020-7489(03)00015-4 12834931

[32] Lugo-Agudelo LH, Cruz Sarmiento KM, Spir Brunal MA, Velasquez Correa JC, Posada Borrero AM, Fernanda Mesa Franco L, et al. Adaptations for rehabilitation services during the COVID-19 pandemic proposed by scientific organizations and rehabilitation professionals. J Rehabil Med 2021; 53: jrm00228. 10.2340/16501977-2865 34427688 PMC8638723

[33] Gilam G, Sturgeon JA, You DS, Wasan AD, Darnall BD, Mackey SC. Negative affect-related factors have the strongest association with prescription opioid misuse in a cross-sectional cohort of patients with chronic pain. Pain Med 2020; 21: e127–e138. 10.1093/pm/pnz249 31617916 PMC7049262

[34] Asmundson GJ, Coons MJ, Taylor S, Katz J. PTSD and the experience of paIn: research and clinical implications of shared vulnerability and mutual maintenance models. Can J Psychiatry 2002; 47: 930–937. 10.1177/070674370204701004 12553128

[35] Gilam G, Gross JJ, Wager TD, Keefe FJ, Mackey SC. What is the relationship between pain and emotion? Bridging constructs and communities. Neuron 2020; 107: 17–21. 10.1016/j.neuron.2020.05.024 32562660 PMC7578761

[36] Simic K, Savic B, Knezevic NN. Pain catastrophizing: how far have we come. Neurol Int 2024; 16: 483–501. 10.3390/neurolint16030036 38804476 PMC11130925

[37] Rogers AH, Farris SG. A meta-analysis of the associations of elements of the fear-avoidance model of chronic pain with negative affect, depression, anxiety, pain-related disability and pain intensity. Eur J Pain 2022; 26: 1611–1635. 10.1002/ejp.1994 35727200 PMC9541898

[38] Cuff L, Fann JR, Bombardier CH, Graves DE, Kalpakjian CZ. Depression, pain intensity, and interference in acute spinal cord injury. Top Spinal Cord Inj Rehabil 2014; 20: 32–39. 10.1310/sci2001-32 24574820 PMC3919692

[39] Finstad J, Roise O, Clausen T, Rosseland LA, Havnes IA. A qualitative longitudinal study of traumatic orthopaedic injury survivors’ experiences with pain and the long-term recovery trajectory. BMJ Open 2024;14: e079161. 10.1136/bmjopen-2023-079161 PMC10806614 38191252

[40] Hamina A, Odsbu I, Borchgrevink PC, Chen LC, Clausen T, Espnes KA, et al. Cohort description: preventing an opioid epidemic in Norway – focusing on treatment of chronic pain (POINT) – A national registry-based study. Clin Epidemiol 2022; 14: 1477–1486. 10.2147/CLEP.S382136 36523790 PMC9744863

[41] Engel S, Rosseland LA, Smits M, Skurtveit SO, Gran JM, Nordseth T, et al. Population-based multistate modelling of peritraumatic opioid use among trauma patients from the Norwegian Trauma Registry. Br J Anaesth 2025; 135: 390–400. 10.1016/j.bja.2025.05.008 40615331 PMC12308083

[42] Sun Y, Wu Y, Fan S, Dal Santo T, Li L, Jiang X, et al. Comparison of mental health symptoms before and during the covid-19 pandemic: evidence from a systematic review and meta-analysis of 134 cohorts. BMJ 2023; 380: e074224. 10.1136/bmj-2022-074224 36889797 PMC9992728

